# The immunologic outcomes and adverse events of COVID-19 vaccine booster dose in immunosuppressed people: A systematic review

**DOI:** 10.1016/j.pmedr.2024.102778

**Published:** 2024-05-31

**Authors:** SeyedAhmad SeyedAlinaghi, Mohsen Dashti, Arian Afzalian, Haleh Siami, Afsaneh Ghasemzadeh, Sanaz Varshochi, Sahar Nooralioghli Parikhani, Masoomeh Fathi Amrollah, Anahid Nourian, Esmaeil Mehraeen, Omid Dadras

**Affiliations:** a Iranian Research Center for HIV/AIDS, Iranian Institute for Reduction of High-Risk Behaviors, Tehran University of Medical Sciences, Tehran, Iran; b Department of Radiology, Tabriz University of Medical Sciences, Tabriz, Iran; c School of Medicine, Tehran University of Medical Sciences, Tehran, Iran; d School of Medicine, Islamic Azad University, Tehran, Iran; e Department of Pharmacotherapy, Faculty of Pharmacy, Tehran University of Medical Sciences, Tehran, Iran; f Department of Health Information Technology, Khalkhal University of Medical Sciences, Khalkhal, Iran

**Keywords:** COVID-19, SARS-CoV-2, Booster dose, Adverse events, Immunologic outcomes, Immunogenicity

## Abstract

**Introduction:**

This study examines the efficacy and safety of three COVID-19 booster vaccines including mRNA-based vaccines (BNT162b2 (BioNTech/Pfizer) and/or mRNA-1273 (Moderna)), Non-Replicating Viral-Vector vaccines (ChAdOx1 nCoV-19 vaccine (AstraZeneca) and/or Ad26. COV2.S (Johnson & Johnson)), and Protein Subunit vaccine (SpikoGen) in immunosuppressed patients.

**Methods:**

Relevant articles were systematically searched using medical subject heading (MeSH) and keywords “COVID‐19” and “booster dose” or “booster vaccine” or ‘’fourth dose” in the online databases of PubMed, Embase, Scopus, and Web of Science. To identify eligible studies, a two-phase screening process was implemented. Initially, three researchers evaluated the studies based on the relevancy of the title and abstract.

**Results:**

A total of 58 studies met the inclusion criteria and were included in this review. The findings suggest that booster doses offer greater protection against the disease than the primary dose. The study also compared various vaccine types, revealing that viral vector and nucleic acid vaccines outperformed inactivated vaccines. Results indicated that individuals receiving booster doses experienced superior outcomes compared to those without boosters. Vaccination against COVID-19 emerged as the most effective preventive measure against infection and symptom severity. Elevated antibody levels post-booster dose vaccination in the population signaled robust immune responses, underscoring the benefits of supplementary vaccine doses.

**Conclusion:**

This systematic review highlights preliminary evidence supporting the immunologic outcomes and safety of COVID-19 vaccine boosters in enhancing immune responses against SARS-CoV-2. However, further research is needed to determine optimal timing intervals between primary vaccination series and boosters while considering global equity issues and variant-specific considerations.

## 1 Introduction

There have been millions of mortalities and morbidities all over the world since the COVID-19 pandemic (David et al., 2022, Guven et al., 2023, SeyedAlinaghi et al., 2022). Although the disease has affected all groups of people, those with chronic comorbidities are among the most vulnerable individuals (Guven et al., 2023, Mehraeen et al., 2022). Certain populations (Balsby et al., 2022) including solid organ transplant recipients (SOTR) such as those who have undergone kidney (Affeldt et al., 2022, Brandstetter et al., 2022, Hod et al., 2022, Schrezenmeier et al., 2022, Thotsiri et al., 2022), liver (Davidov et al., 2022, Sriphoosanaphan et al., 2022), lung (Catry et al., 2022) and heart transplants (Peled et al., 2022) as well as patients with conditions like immune-mediated inflammatory diseases and multiple sclerosis who receive immunosuppressive treatments (Bjørlykke et al., 2023, Smetanova et al., 2022, Wroński et al., 2022), face greater disease severity. Furthermore, patients diagnosed with various malignancies (Benitez Fuentes et al., 2022, Diamantopoulos et al., 2022, Goldwater et al., 2023, Ollila et al., 2022, Storti et al., 2022, Tanzilli et al., 2022, Terpos et al., 2022) or immune deficiencies (Fidler et al., 2023, Gianserra et al., 2022, Kling et al., 2023) experience a more severe course of illness due to a combination of their immunosuppressive medications and the nature of their underlying condition. The use of immunosuppressive medications is significantly associated with the severity of COVID-19 and increased hospitalizations (Kontopoulou et al., 2022, Shapiro Ben David et al., 2022). Additionally, patients with conditions that impair the immune system, such as those undergoing hemodialysis or peritoneal dialysis, are also at a heightened risk for severe COVID-19 outcomes (Affeldt et al., 2022, Quiroga et al., 2022).

Immunity to SARS-CoV-2 in individuals and populations arises from either prior infection or vaccination, mediated by humoral and cellular immune responses (Thotsiri et al., 2022, Hod et al., 2023). The effectiveness of COVID-19 vaccines in preventing SARS-CoV-2 infection and diminishing the severity of the disease has been established (Lin et al., 2022, Dadras et al., 2022). Since the immune response subsides after several months after vaccination, especially with the emergence of new fast spreading variants of concern (VOCs) like Omicron variant (Peled et al., 2022, Kling et al., 2023), periodic heterologous or homologous booster doses are essential to ensure efficient and long-lasting protection against COVID-19 (Sriphoosanaphan et al., 2022, Catry et al., 2022, Guven et al., 2023, Peled et al., 2022).

Immune responses among immunosuppressed patients have been demonstrated to be less robust than in the general population (Balsby et al., 2022, Schrezenmeier et al., 2022, Kontopoulou et al., 2022, Lamacchia et al., 2022, Piñana et al., 2023, Yang et al., 2022, Mehraeen et al., 2022). Moreover, these patients experience a more rapid decline in both antibody response and cellular immunity compared to healthy individuals (Affeldt et al., 2022, Brandstetter et al., 2022), leaving them with diminished protection against SARS-CoV-2 infection. Therefore, impaired immune response necessitates repeated booster doses particularly in vulnerable and immunocompromised patients (Affeldt et al., 2022, Yang et al., 2022, Lin et al., 2022). Furthermore, some studies have demonstrated that decreasing T-cell (cellular) immunity is not parallel to waning antibody (humoral) response after the booster dose (Lin et al., 2022, Torres et al., 2022) and reduction in disease severity is not completely based on measured humoral response (Hod et al., 2023); but the exact roles are not completely known (Wroński et al., 2022). Although these groups of patients were excluded from clinical trials for marketing authorization, based on findings of different studies administration of a third (Goldwater et al., 2023, Feingold et al., 2022) and in some cases a fourth dose (Davidov et al., 2022, Fendler et al., 2022) of vaccine is recommended in immunocompromised patients. The safety of SARS-CoV-2 vaccines has also been evaluated in different groups of these patients (Brandstetter et al., 2022, Sriphoosanaphan et al., 2022, Peled et al., 2022, Bjørlykke et al., 2023, Wroński et al., 2022, Tanzilli et al., 2022, Gianserra et al., 2022, Peled et al., 2022, Crane et al., 2023) (Davidov et al., 2022, Smetanova et al., 2022, Tanzilli et al., 2022, Terpos et al., 2022, Kling et al., 2023, Lin et al., 2022, Benotmane et al., 2022, Gavriatopoulou et al., 2022, Kim et al., 2022).

In this study, we aimed to review the immunologic outcomes and safety of three types of COVID-19 booster vaccines including mRNA-based vaccines (BNT162b2 (Pfizer-BioNTech) and/or mRNA-1273 (Moderna-NIAID)), Non-Replicating Viral-Vector vaccines (ChAdOx1 nCoV-19 (Oxford-AstraZeneca) and/or Ad26.COV2-S (Johnson&Johnson)), and Protein Subunit vaccine (SpikoGen (Vaxine)) in immunosuppressed patients (see Table 1).

## 2 Methods

### 2.1 Study objects

**Fig. 1:**
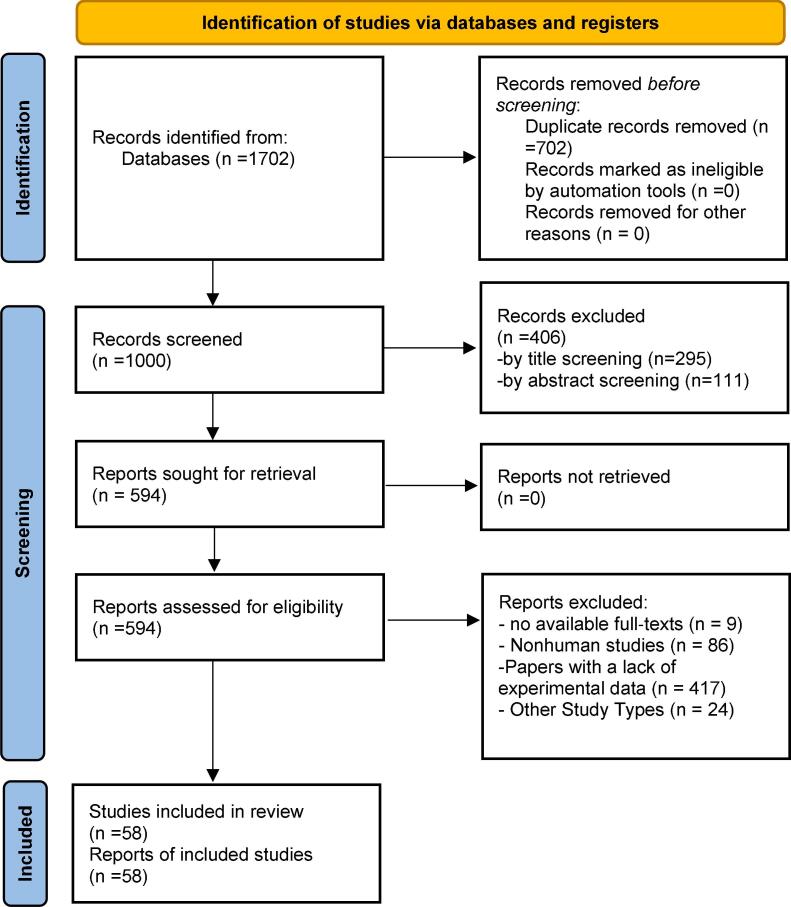
PRISMA 2020 flow diagram of the study retrieval process.

The primary aim of this study was to investigate the indications for administering COVID‐19 booster vaccines in immunocompromised population, particularly those with medical conditions such as organ transplantation, cancer, HIV, etc. Additionally, the study discussed the need for the necessity of administering booster doses every 6 or 12 months and identifying the subpopulation that would derive the most benefit. To ensure adherence to reporting standards, this study followed the Preferred Reporting Items for Systematic Reviews and Meta-Analyses (PRISMA) checklist (Fig. 1).

### 2.2 Data sources

Relevant articles were systematically searched using medical subject heading (MeSH) and keywords “COVID‐19” and “booster dose” or “booster vaccine” or ‘’fourth dose” in the online databases of PubMed, Embase, Scopus, and Web of Science. All the relevant literature published between January 2022 and February 2023 was retrieved and further screened using EndNote™ 20.2 reference manager (Clarivate Analytics©).

### 2.3 Study selection and inclusion/exclusion criteria

We conducted a two‐phase screening process in order to select the eligible studies. Initially, three researchers evaluated the studies based on the relevancy of the title and abstract. Subsequently, the same researchers reviewed the full texts of the remaining studies and selected the most suitable ones according to the inclusion criteria. Any disagreements among the researchers were resolved by involving another independent researcher to address inconsistencies in the results. We included peer‐reviewed original articles that examined indications of COVID-19 booster dose or vaccines. The selected articles were cross‐examined by other researchers to avoid duplication.

The exclusion criteria were as follows:

Literature with no published/available full‐texts including conference papers, abstracts, preprints, research letters, and research notes;

Nonhuman studies or experiments of any kind like in vitro studies, animal trials, or literature without justifying details;

Non-original studies including reviews, systematic reviews, meta‐analyses, and opinions;

## 3 Case reports

### 3.1 Data extraction

Four researchers summarized and extracted the following information from the full-text of included publications: the first author's ID (Reference), year, and type of publication (e.g., clinical trial), country where the study was conducted, sample size, target population, type or name of the vaccine, serious adverse events, side effects, and the rationale for administering the booster dose. This data was then compiled into a specifically designed sheet and organized into tables for easy comparison and data presentation.

### 3.2 Quality/risk of bias assessment

To ensure the authenticity, reliability, and quality of the outcomes, we utilized the Newcastle–Ottawa Scale (NOS) to assess the quality of the studies. The NOS consists of three items: selection, comparability, and exposure/outcome. Each item is graded maximum scores of 4, 2, and 3 respectively. By summing up these values, a maximum score of 9 is allocated to each individual study (Table 2).

**Table 2:** Newcastle-Ottawa Scale (NOS) bias risk assessment of the study.

**ID**	**1st Author (Reference)**	**Selection (out of 4)**	**Comparability (out of 2)**	**Exposure/Outcome (out of 3)**	**Total** **(Out of 9)**
1	(Affeldt et al., 2022)	3	2	3	8
2	(Balsby et al., 2022)	2	1	2	5
3	(Benitez Fuentes et al., 2022)	2	2	3	7
4	(Benotmane et al., 2022)	2	1	1	4
5	(Bjørlykke et al., 2023)	3	1	3	7
6	(Brandstetter et al., 2022)	4	2	3	9
7	(Busà et al., 2022)	3	2	3	8
8	(Cassaniti et al., 2022)	4	1	2	7
9	(Catry et al., 2022)	3	2	3	8
10	(Costard-Jäckle et al., 2022)	3	2	3	8
11	(Crane et al., 2023)	2	1	2	5
12	(Davidov et al., 2022)	3	1	3	7
13	(Diamantopoulos et al., 2022)	2	1	1	4
14	(Feingold et al., 2022)	3	1	3	7
15	(Fendler et al., 2022)	4	2	3	9
16	(Fidler et al., 2023)	3	2	3	8
17	(Fiorino et al., 2022)	3	2	3	8
18	(Gavriatopoulou et al., 2022)	2	1	2	5
19	(Gianserra et al., 2022)	2	0	3	5
20	(Goldwater et al., 2023)	2	1	1	4
21	(Guven et al., 2023)	3	1	3	7
22	(Hod et al., 2023)	2	1	2	5
23	(Hod et al., 2022)	4	2	2	8
24	(Karaba et al., 2022)	2	1	3	6
25	(Kim et al., 2022)	2	0	3	5
26	(Kling et al., 2023)	2	1	2	5
27	(Kontopoulou et al., 2022)	4	2	3	9
28	(Lamacchia et al., 2022)	2	1	2	5
29	(Lin et al., 2022)	4	2	2	8
30	(Lin et al., 2022)	2	1	2	5
31	(Madelon et al., 2022)	4	2	3	9
32	(Maniscalco et al., 2022)	2	1	3	6
33	(Moser et al., 2022)	2	0	3	5
34	(Nafar et al., 2022)	2	1	1	4
35	(Ollila et al., 2022)	3	1	3	7
36	(Peled et al., 2022)	4	2	3	9
37	(Peled et al., 2022)	4	2	3	9
38	(Perrier et al., 2022)	2	1	2	5
39	(Piñana et al., 2023)	4	1	3	8
40	(Pulvirenti et al., 2022)	3	2	2	7
41	(Pulvirenti et al., 2023)	4	1	3	8
42	(Quiroga et al., 2022)	3	2	2	7
43	(Schrezenmeier et al., 2022)	4	2	3	9
44	(Shashar et al., 2022)	4	2	3	9
45	(Shen et al., 2022)	2	1	2	5
46	(Shapiro Ben David et al., 2022)	3	2	3	8
47	(Smetanova et al., 2022)	2	2	1	5
48	(Sriphoosanaphan et al., 2022)	3	1	3	7
49	(Storti et al., 2022)	4	2	3	9
50	(Su et al., 2022)	3	2	3	8
51	(Tanzilli et al., 2022)	2	1	2	5
52	(Tenforde et al., 2022)	4	1	3	8
53	(Terpos et al., 2022)	2	2	1	5
54	(Thotsiri et al., 2022)	3	1	3	7
55	(Torres et al., 2022)	2	2	3	7
56	(Verdier et al., 2022)	3	2	3	8
57	(Wroński et al., 2022)	4	2	3	9
58	(Yang et al., 2022)	3	2	3	8

## 4 Results

The primary search strategy of online databases yielded 1702 papers. After the first review 702 duplicate records were found and excluded. The remaining 1000 articles underwent title and abstract screening by two independent researchers leading to extraction of 81 articles for full text assessment. Finally, 58 articles met the eligibility criteria and were involved in the final review. The PRISMA Flow Diagram (Fig. 1) demonstrates the thorough detail of our selection process.

### 4.1 Characteristics of the included studies

All studies were conducted in 2022 (n = 51) and 2023 (n = 7). Eleven studies were carried out in the USA, 10 in Italy, and seven in Israel. Spain and Greece each had four, France and Germany each had three, and Austria, Switzerland, and Thailand each had two involved papers. Denmark, Norway, Belgium, UK, Turkey, South Korea, Taiwan, Iran, Czech, and Poland each had one included article. In regards to the type of studies, the most common type was cohort studies (n = 25), followed by; prospective observational (n = 17), retrospective observational (n = 7), RCT (n = 3), Case-Control (n = 2), cross-sectional (n = 2), and prospective single arm trial (n = 2). A total of 16,476 individuals were involved and investigated in all 58 studies and the study population of included papers ranged between 8 and 4283 subjects (Lamacchia et al., 2022, Shen et al., 2022).

### 4.2 Immunocompromised groups

In our review six studies had investigated the safety and efficacy of SARS-CoV-2 booster among an extended spectrum of immunocompromised patients (Kontopoulou et al., 2022, Shapiro Ben David et al., 2022, Lin et al., 2022, Yang et al., 2022, Shen et al., 2022, Tenforde et al., 2022) while in the remaining 52 studies, researchers had studied on specific immunocompromised subgroups as follows; transplant-recipients (n = 21) (Balsby et al., 2022, Affeldt et al., 2022, Brandstetter et al., 2022, Hod et al., 2022, Schrezenmeier et al., 2022, Thotsiri et al., 2022, Davidov et al., 2022, Sriphoosanaphan et al., 2022, Catry et al., 2022, Peled et al., 2022, Hod et al., 2023, Peled et al., 2022, Fendler et al., 2022, Crane et al., 2023, Benotmane et al., 2022, Busà et al., 2022, Cassaniti et al., 2022, Costard-Jäckle et al., 2022, Karaba et al., 2022, Nafar et al., 2022, Perrier et al., 2022), hematologic malignancies (n = 9) (Diamantopoulos et al., 2022, Goldwater et al., 2023, Ollila et al., 2022, Storti et al., 2022, Terpos et al., 2022, Piñana et al., 2023, Fendler et al., 2022, Fiorino et al., 2022, Gavriatopoulou et al., 2022), solid tumors and cancers (n = 5) (Benitez Fuentes et al., 2022, Tanzilli et al., 2022, Guven et al., 2023, Kim et al., 2022, Su et al., 2022), dialysis (n = 4) (Affeldt et al., 2022, Quiroga et al., 2022, Shashar et al., 2022, Verdier et al., 2022), HIV (n = 4) (Fidler et al., 2023, Gianserra et al., 2022, Kling et al., 2023, Lamacchia et al., 2022), primary immunodeficiencies (n = 3) (Lin et al., 2022, Pulvirenti et al., 2022, Pulvirenti et al., 2023); immune-mediated inflammatory diseases (n = 3) (Bjørlykke et al., 2023, Smetanova et al., 2022, Wroński et al., 2022), multiple sclerosis (n = 2) (Madelon et al., 2022, Maniscalco et al., 2022), and anti-CD20 treatment (n = 2) (Torres et al., 2022, Moser et al., 2022).

### 4.3 Vaccine types

In total three types of SARS-CoV-2 vaccines were used as booster doses including mRNA-based vaccines (n = 72) including BNT162b2 (BioNTech/Pfizer) and/or mRNA-1273 (Moderna), Non-Replicating Viral-Vector vaccines (n = 11) including ChAdOx1 nCoV-19 vaccine (AstraZeneca) and/or Ad26. COV2.S (Johnson & Johnson), and Protein Subunit vaccine (n = 1) including SpikoGen vaccine. BNT162b2 vaccine was the most commonly used booster (n = 47) followed by mRNA-1273 (n = 22), ChAdOx1 nCoV-19 (n = 8), and Ad26. COV2.S (n = 3), and SpikoGen (n = 1).

### 4.4 Vaccine doses and efficacy assessment

In all included studies, the efficacy of the 3rd dose vaccination was investigated, with 15 articles also assessing the efficacy of a 4th dose. Among the 58 articles included to evaluate booster vaccine efficacy, 54 examined the immune system humoral (n = 50) and/or cellular (n = 17) responses through six distinct methods. The most common utilized methods involved measuring blood titers of SARS-CoV-2 Anti-Spike or SARS-CoV-2 Anti-receptor binding domain (RBD) IgG Antibody, employing in 49 articles, and plasma neutralizing activity assays or Neutralizing antibody titers (NAbs), used in 18 papers. Other methods included Spike-specific T and/or B cell response (n = 12), SARS-CoV-2 nucleocapsid IgG antibodies (n = 7), interferon-γ (IFN-γ) levels (n = 6), and CD4^+^ and/or CD8^+^ T-cell counts (n = 3). The remaining four papers utilized infection and/or mortality rates to assess the efficacy of booster vaccinations.

### 4.5 Vaccine response assessment onset and the interval between vaccine doses

A total of 53 articles specified the efficacy assessment onset, after the booster dose administration. The vaccine response evaluation onset ranged between a low of seven days up to 12 months after the booster dose (37, 38). Additionally, 42 papers reported the time interval between the primary two-dose vaccination course and 3rd and/or 4th boosting doses, the administration interval between the 2nd dose and the 3rd dose ranged between a low of 28 days to a peak of one year (Fidler et al., 2023, Tenforde et al., 2022), and the interval between 3rd and 4th dose administration fell between a range of 21 to 201 days (Cassaniti et al., 2022, Perrier et al., 2022).

### 4.6 Adverse events

Overall, 13 papers reported adverse events after the COVID-19 booster vaccination (Brandstetter et al., 2022, Hod et al., 2022, Davidov et al., 2022, Sriphoosanaphan et al., 2022, Bjørlykke et al., 2023, Wroński et al., 2022, Tanzilli et al., 2022, Gianserra et al., 2022, Hod et al., 2023, Peled et al., 2022, Feingold et al., 2022, Crane et al., 2023, Nafar et al., 2022). The majority reported minor local and/or systemic complications that had resolved, except one paper among KTRs that had reported one case of acute kidney rejection after 2 weeks of the 3rd dose, and one other case of rejection (with history of non-adherence to immunosuppression) after four months of the 2nd dose (Brandstetter et al., 2022).

### 4.7 Transplant patients

The most common studied population were transplant recipients; 10 studies were conducted among kidney transplant recipients (KTRs) (Affeldt et al., 2022, Brandstetter et al., 2022, Hod et al., 2022, Schrezenmeier et al., 2022, Thotsiri et al., 2022, Hod et al., 2023, Crane et al., 2023, Benotmane et al., 2022, Cassaniti et al., 2022, Nafar et al., 2022), five were carried out among cardiothoracic transplant patients (Catry et al., 2022, Peled et al., 2022, Peled et al., 2022, Feingold et al., 2022, Costard-Jäckle et al., 2022), four among solid organ transplant cases (Balsby et al., 2022, Busà et al., 2022, Karaba et al., 2022, Perrier et al., 2022), and two among liver transplant individuals (Davidov et al., 2022, Sriphoosanaphan et al., 2022).

## 5 Discussion

In this study, we evaluated the immunologic outcomes and safety of the COVID-19 booster dose against the disease and its side effects. While every dose of the vaccine offers protection, our findings indicate that booster doses are more potent. Among the three vaccine types, the inactivated vaccine was the least effective. Viral vector vaccines and nucleic acid vaccines demonstrated greater efficacy, with one outperforming the other (Xu et al., 2023). We observed that individuals who received the COVID-19 booster dose generally experienced better or equivalent outcomes compared to those who didn't. It remains clear that vaccination against COVID-19 is the most effective strategy for preventing SARS-CoV-2 infection and reducing the severity of symptoms (Xu et al., 2023, Sridhar et al., 2022). This research showcased elevated immunogenicity rates, including heightened SARS-CoV-2 Anti-Spike or SARS-CoV-2 Anti-receptor binding domain (RBD) IgG Antibody titers and increased plasma neutralizing activity or Neutralizing antibody titers (NAbs) post-booster vaccination. Booster doses effectively elevated and maintained these antibody levels | (Nantanee et al., 2022).

In this review of 58 studies, we examine the effects of booster doses and their side effects on various immunosuppressed populations including organ transplant recipients, patients with solid cancers, patients undergoing hemodialysis, people living with HIV, patients with MS, patients with primary immunodeficiency, patients with immune-mediated inflammatory diseases, and patients who are immunocompromised. Our findings underscore the significance of the COVID-19 vaccine's booster doses. The benefits of additional vaccine doses and booster injections are well-established.

## 6 Benefits of a booster dose of COVID-19 vaccine

### 6.1 *Kidney transplant* patients

In a study by Affeldt et al. on KTRs, only 10 out of 29 patients had a measurable humoral immune response after three doses (Affeldt et al., 2022). Another study on 67 KTRs revealed that following the 4th dose, the median anti-RBD titer increased significantly, and the proportion of patients who had neutralizing antibodies against the Delta strain rose from 16 % to 66 % before and after the 4th dose (Benotmane et al., 2022). Brandstetter et al. observed seroconversion rates of 51.54 % after the 2nd dose, 63.95 % after the 3rd dose, and 29.27 % post the 4th dose (Brandstetter et al., 2022). Cassaniti et al. also noted that six months after the 2nd dose, total IgG, SARS-CoV-2 NAbs and Spike-specific T cell response prevalence rates were 45 %, 38 %, and 59.5 %. These rates increased to 53 %, 60 %, and 75.6 % one month after the 3rd dose, respectively (Cassaniti et al., 2022). Another study found that seroconversion among KTRs rose from 56 % in two-dose recipients to 85 % in those administered a 3rd dose (Crane et al., 2023). Hod et al. reported that in their population of KTRs, the response rate rose from 32.3 % before the 3rd dose to 85.9 % after the 3rd dose, and 80.6 % seroconverted and 96.9 % remained positive following the 3rd dose with a significant increase in RBD IgG and Nabs (Hod et al., 2022). In yet another study focusing on KTRs, it was found that the response rate, based on NAb titers, increased from 78.4 % just before the 4th vaccine dose to 94.6 % three weeks afterward (Hod et al., 2023). One single-arm prospective clinical trial on the efficacy of SpikoGen 3rd dose among renal transplant patients found that the seroconversion of neutralizing antibodies was 76 % in the Spikogen group versus 3 % in the placebo group and the seroconversion of RBD IgG was 64 % for SpikoGen group versus 0 % for the placebo group (Nafar et al., 2022). Schrezenmeier et al. pointed out that 76 % of KTRs exhibited anti–S1 domain IgG levels above the threshold for positivity post the 4th dose (Schrezenmeier et al., 2022). Thotsiri et al., in their analysis of 146 KTRs, reported mortality rates of 26 %, 3 %, and 3 % among recipients of 0 to 1 dose, 2 doses, and 3 doses respectively (Thotsiri et al., 2022).

### 6.2 Cardiothoracic transplant patients

Catry et al. studied 49 lung transplant recipients and found that, among those who received the booster dose, the serological response was 32.2 % after 28 days. This third dose resulted in significant increases in both IgG titers and neutralizing antibodies (Catry et al., 2022). Another comprehensive study involving 243 cardiothoracic transplant recipients (228 heart, 14 lungs, 1 heart–lung) showed that 53 % became seropositive after the third dose, with 56 % of these seropositive individuals previously having no detectable IgG titers prior to the booster (Costard-Jäckle et al., 2022). Feingold et al. studied 28 heart transplant patients and observed antibody production after 3rd dose among 57 % of recipients who were negative after their 2nd dose (Feingold et al., 2022). Peled et al. found that in their study among 96 heart transplant patients, the percentage of positive antibody response rose from 23 % prior to 3rd dose to 67 % at 18 days following the 3rd dose, and the 3rd dose induced SARS-CoV-2 neutralization titers to > 9-fold and IgG anti-RBD antibodies > 3-fold of the range achieved after the two primary doses (Peled et al., 2022). Peled et al. also conducted a study on the efficacy of the 4th dose among heart transplant patients found that the detectable Anti-RBD IgG antibodies rose from 61.4 % to 80.7 % before and after the 4th dose, respectively. They also reported that the percentages of NAbs against the wild-type (WT), the Delta, and Omicron variants rose from 48 %, 47 %, and 24 % to 68 %, 66 %, and 49 %, respectively (Peled et al., 2022).

### 6.3 *Solid organ transplant* patients

In a study involving 395 solid organ transplant recipients (SOTR), including kidney, liver, heart, lung, and combined transplants, Balsby et al. reported that the percentage of participants with detectable levels of SARS-CoV-2 spike S1 IgG antibodies increased from 49.4 % after the second dose to 77.9 % after the third dose. Moreover, 47.5 % of those who were seronegative after the second dose became seropositive following the third dose (Balsby et al., 2022). Similarly, Busà et al observed a 3-folds rise in Anti Spike Protein IgG titers after the 4th dose compared to the post-3rd dose among SOTRs (Busà et al., 2022). On the other hand, in the Karaba et al. study the anti-N IgG titers did not significantly differ after the 4th dose, while the Anti-RBD and anti-S seropositivity increased from 56 % to 84 % and from 68 % to 88 %, respectively (Karaba et al., 2022). Perrier et al., in a large study among 825 SORTs found that proportion of participants with a strong humoral response increased significantly with the number of vaccine doses as flows: 10.6 % after the 1st dose, 35.1 % after the 2nd, 48.5 % after the 3rd, and 65.1 % after the 4th dose (Perrier et al., 2022).

### 6.4 Liver transplant patients

Davidov et al., in their study on 73 liver transplant adults found statistically significant increases in RBD IgG and Omicron BA.1 and BA.2 Nab titers when comparing 3rd and 4th doses. They found that breakthrough infections occurred in 18 % of 4th dose recipients vs. 30.4 % of 3rd dose recipients (Davidov et al., 2022). One study among 89 liver transplant patients also reported that seroconversion was observed in 81.3 % of liver transplant patients receiving ChAdOx1/ChAdOx1/mRNA-1273 and in 94.7 % of those receiving ChAdOx1/BNT162b2/mRNA-1273 (Sriphoosanaphan et al., 2022).

### 6.5 Blood cancer patients

Nine papers studied the booster vaccination efficacy among people with hematological cancers or dysplasia (Diamantopoulos et al., 2022, Goldwater et al., 2023, Ollila et al., 2022, Storti et al., 2022, Terpos et al., 2022, Piñana et al., 2023, Fendler et al., 2022, Fiorino et al., 2022, Gavriatopoulou et al., 2022). Diamantopoulos et al. in their study of 39 chronic lymphocytic leukemia (CLL) patients, observed that the seroconversion rate rose from 28.2 % to 64.1 % after the 3rd dose and it was higher among treatment-naïve patients (Diamantopoulos et al., 2022). One study among 80 patients with hematological cancers reported that after 3rd dose, 62 %, 87 %, and 72 % of patients had detectable NAbs against Omicron BA.1, WT, and Delta, respectively. They also found that following the 4th dose, the detectable NAbT rate against Omicron BA.1, WT, and Delta rose to 79 %, 98 %, and 78 %, respectively (Fendler et al., 2022). One study, conducted among myelofibrosis patients, found that the 3rd dose significantly increased the anti-spike IgG titers, reaching antibody levels in both myelofibrosis patients and healthy controls (Fiorino et al., 2022). Gavriatopoulou et al., in their study among 58 patients with Waldenstrom Macroglobulinemia (WM), CLL, and Non-Hodgkin Lymphoma (NHL), observed that the booster dose of both Pfizer and AstraZeneca vaccines resulted in the lower level compared to healthy individuals (Gavriatopoulou et al., 2022). One study among multiple myeloma (MM) patients reported that the 3rd dose vaccination increased antibody levels and achieved higher levels than peak levels after the first two doses (Storti et al., 2022). A similar study among MM and pre-malignant monoclonal gammopathies showed that heterologous booster immunization improves SARS-CoV-2 spike humoral and cellular responses in newly diagnosed MM patients and in most, but not all, MM patients with relapsed-refractory disease (Storti et al., 2022). One large study among 378 patients with hematologic malignancy reported that among initial non-responders to primary two doses, seroconversion after the 3rd dose occurred in 56 % of patients (The seroconversion rate after the booster was similar for patients on (53 %) and off (58 %) active therapy) (Ollila et al., 2022). Piñana et al., in their study of 1551 patients with hematological disorders, found that 28.5 % of patients developed SARS-CoV-2 infection after full primary vaccination and before the booster dose, whereas 12.8 % of those who received the 3rd dose and 5.8 % of those who received the 4th dose was infected with SARS-CoV-2 (Piñana et al., 2023). Terpos et al. in their study among B‐cell malignancy patients reported that the percentage of patients with NAb > 50 % rose from 23.5 % to 77.9 % before and after the 3rd dose (Terpos et al., 2022).

### 6.6 Patients with solid tumors and/or metastasis

Five studies investigated the efficacy of COVID-19 booster doses among patients with solid tumors (Benitez Fuentes et al., 2022, Tanzilli et al., 2022, Guven et al., 2023, Kim et al., 2022, Su et al., 2022). Benitez et al. observed higher antibody levels when comparing the antibody responses after the 1st to the 3rd and the 2nd to the 3rd vaccine doses (Benitez Fuentes et al., 2022). According to a cohort study conducted in 2023 by Tan et al., there is evidence of the clinical effectiveness of mRNA-based vaccines against COVID-19 in cancer patients. The study also mentioned that during both the delta and omicron waves, there was a significant decrease in the incidence rate ratio for COVID-19 hospitalization and severe disease in the 3-dose and 4-dose groups compared to the 2-dose group (Tan et al., 2023). Guven et al. reported a rise in seroconversion rates, ranging from 46.5 % to 88.5 %, following the 3rd dose, and also observed a significant boost in antibody titers after this dose (Guven et al., 2023). In one study among 40 patients with early breast cancer, the breast cancer group had a 2.5-fold rise in SARS-CoV-2 spike IgG response after the 3rd dose, compared to a 4.0-fold increase in the healthy control group (Kim et al., 2022). In another study among 51 patients with metastatic solid malignancies, a significant increase in the anti-S antibody levels and SARS-CoV-2-specific T-cells was observed after the booster vaccination (Su et al., 2022). Tanzilli et al, in their study among 112 primary brain tumor patients found that in total among all vaccine recipients only 4 cases were infected by the SARS-CoV-2 without symptoms (Tanzilli et al., 2022). According to a study by Scherer et al., of the 4 756 102 doses that were given to the study population between September 15, 2021, and November 11, 2022, 4 462 301 (93.8 %) were monovalent mRNA vaccines, 76 247 (1.6 %) were bivalent mRNA vaccines, and the remaining 217 554 (4.6 %) were non-mRNA vaccines. In cancer patients receiving active treatment, a third dose significantly reduced the chance of severe COVID-19, albeit less so than in matched controls. In contrast to those infected before 60 days, those infected more than 150 days after their third dosage did not exhibit a statistically greater risk of hospitalization or serious illness, indicating a prolonged protective effect. Five months after the third dose, Singapore started giving out the fourth, becoming one of the first countries in the world to do so (Scherer et al., 2022). Further research, published by Lee et al., showed a longitudinal correlation between the duration since the last vaccination and the clinical outcomes of COVID-19 infection in cancer patients at a representative population level. Vaccine effectiveness at three to six months was found to be lower in the cancer cohort (47·0%, 46·3–47·6) compared to the control group (61·4%, 61·4–61·5) (Lee et al., 2022).

### 6.7 Dialysis patients

Four studies investigated the efficacy of COVID-19 booster vaccination among patients undergoing dialysis; three among hemodialysis patients (Affeldt et al., 2022, Shashar et al., 2022, Verdier et al., 2022) and one among peritoneal dialysis patients (Quiroga et al., 2022). The Anti-SARS-CoV-2 IgG antibody titers in all four studies increased significantly after the 3rd dose. Affeldt et al. reported that four doses of the Moderna vaccine resulted in higher IgG levels than four doses of Pfizer vaccine among hemodialysis patients (Affeldt et al., 2022). Shashar et al. found that humoral response after the booster was positively correlated with albumin and inversely correlated with C-reactive protein (CRP) (Shashar et al., 2022). Verdier et al. Also stated that the mean body mass index and albumin levels were significantly higher among responders after two doses of vaccine (Verdier et al., 2022). Quiroga et al., in their study on 164 peritoneal dialysis patients, found that only a 3rd dose caused significantly higher anti-spike antibody titers when compared to two-dose recipients while the antibody increase after the 4th dose was not statistically significant (Quiroga et al., 2022).

### 6.8 *People living with HIV (*PLWH*)*

Four studies investigated the booster dose efficacy among PLWH (Fidler et al., 2023, Gianserra et al., 2022, Kling et al., 2023, Lamacchia et al., 2022). One RCT among 43 PLWH found that the levels of Anti-SARS-CoV-2 spike IgG and CD4^+^ SARS-CoV-2 were significantly higher after the 3rd dose recipients compared to baseline levels (Fidler et al., 2023). Gianserra et al., in their study of 42 PLWH, observed a rise in both CD4^+^ and CD8^+^ T-cell median counts after the 3rd dose (Gianserra et al., 2022). Kling et al. found that there was no significant difference in anti-spike IgG levels or viral neutralization responses against WT, Delta, and Omicron variants between PLWH and controls (Kling et al., 2023). Lamacchia et al found that in their study population the Anti-spike IgG levels were above the cut-off value for all PLWH at all timepoints (Lamacchia et al., 2022).

### 6.9 *Multiple* sclerosis *(MS) and/or patients undergoing Anti-CD20 treatment*

Four studies were conducted among MS and/or patients undergoing Anti-CD20 treatment (Torres et al., 2022, Madelon et al., 2022, Maniscalco et al., 2022, Moser et al., 2022). Madelon et al., in their study of 20 MS patients treated with anti-CD20 drugs, reported that the levels of cytotoxic T cells, specific for the vaccine strain, Delta, and Omicron variants, increased after the 3rd dose (Madelon et al., 2022). One study conducted among 65 MS subjects undergoing different disease-modifying therapies reported that 100 % of healthy controls and 97 % of disease-modifying therapies-treated MS patients were seropositive after the 3rd dose (Maniscalco et al., 2022). One study of 15 MS patients on anti-CD20 treatments found that the antibody levels were significantly higher among patients who only received the booster dose amid B cell depletion comparing to patients vaccinated during a continuous state of B cell depletion (Moser et al., 2022). Torres et al. reported an increase of 1.53-fold in SARS-CoV-2 Specific IgGs in rituximab-treated patients compared to a 2.47-fold increase in healthy donors one month after receiving the booster dose, they also noted that 100 % of healthy controls and 88.9 % of rituximab-treated patients showed SARS-CoV-2 IgGs after the booster dose (Torres et al., 2022).

### 6.10 Primary immunodeficiency patients

Three papers studied booster dose efficacy among primary immunodeficient patients; one among primary antibody deficiency patients (Lin et al., 2022), one among patients with Common Variable Immune Deficiency (CVID) (Pulvirenti et al., 2022), and one among with 22q11.2 deletion syndrome patients (Pulvirenti et al., 2023). Observed that COVID-19-naïve individuals with primary antibody deficiencies exhibited SARS-CoV-2-specific B and T cell responses after a booster vaccination and developed Omicron-specific memory B cells (Lin et al., 2022). Pulvirenti et al. reported that among CVID patients, the percentage of patients with measurable anti-S1 IgG dose rose from 20 % to 64 % after the 3rd dose, they also in another study among 16 patients with 22q11.2 deletion syndrome found that anti-S1 antibody titers decreased over time while they were significantly boosted by the 3rd dose (Pulvirenti et al., 2022, Pulvirenti et al., 2023).

### 6.11 *Patients with immune-*mediated *inflammatory diseases*

Three papers studied the booster dose efficacy among patients with immune-mediated inflammatory diseases. One study was conducted among a large group of patients with one of the following conditions: rheumatoid arthritis, spondyloarthritis, psoriatic arthritis, Crohn's disease, or ulcerative colitis (Bjørlykke et al., 2023). Another study investigated patients with spondyloarthritis treated with interleukin-17 (IL-17) and/or tumor necrosis factor-alpha (TNFa) Inhibitors (Smetanova et al., 2022), and one was carried out among patients with inflammatory arthritis (Wroński et al., 2022). Bjørlykke et al. reported that among the 536 patients with inflammatory diseases who received the 4th dose, anti-RBD antibody titers were significantly higher after the 4th dose than the 3rd dose, but were significantly lower when comparing to the healthy control group vaccinated with three doses (Bjørlykke et al., 2023). Smetanova et la. in their study among 15 spondyloarthritis reported an increase in SARS-CoV-2 specific antibody titers after the 3rd dose in both TNF-a and/or IL-17 recipients (Smetanova et al., 2022). One study among 49 inflammatory arthritis patients, reported an increase in humoral response among all participants but the IgG levels rose more significantly among healthy control group compared to patients. In addition, the cellular response was significantly lower both before and after the booster dose among inflammatory arthritis patients when compared to controls (Wroński et al., 2022).

### 6.12 *Immunocompromised* patients

Finally, six papers studied immunocompromised subject on a broad scale. Kontopoulou et al. reported that among immunocompromised individuals, the overall IgG titers 4 weeks after 3rd dose increased by more than 35-folds (Kontopoulou et al., 2022). Lin et al. also found that immunocompromised patients had similar anti-SARS-CoV-2 spike IgG titers 4 weeks after booster dose compared with healthy participants aged ≤ 50 years, and specified that only participants with autoimmune diseases and receiving hydroxychloroquine, low-dose steroid, methotrexate, and/or sulfasalazine had numerically lower anti-SARS-CoV-2 spike IgG titers 4 weeks after booster vaccination compared to those without (Lin et al., 2022). Shen et al. conducted a large study among 4283 individuals under immunosuppressants, and reported that fully vaccinated immunosuppressed subjects who had a booster dose had a lower incidence of SARS-CoV-2 infection compared to those without a booster (Shen et al., 2022). Another study stated that one month after the 3rd dose, 80 % of immunocompromised and 100 % of immunocompetent subjects showed an antibody response. Additionally, post the 3rd dose, IgG titers increased 7.83-fold and 2.40-fold in the immunocompromised and immunocompetent groups, respectively, compared to after the 2nd dose (Shapiro Ben David et al., 2022). Tenforde et al. reported that among the immunocompromised patients, vaccine was more effective among 3-dose recipients (20 % COVID-19 case-patients) compared with 2-dose recipients (30 % COVID-19 case-patients) (Tenforde et al., 2022). In a study by Yang et al. on immunocompromised patients, results showed that post the initial 2 doses, 62 % had positive anti-S1 IgG and 71 % tested positive for SARS-CoV-2 interferon gamma release assay (IGRA). Following the booster, these figures rose to 69 % and 73 % respectively (Yang et al., 2022).

### 6.13 General population

Additionally, several studies support the efficacy of booster dosage vaccinations in the general public. According to a study by Bruxvoort et al., the mRNA-1273 vaccination protects against SARS-CoV-2 infection. The additional dose of the COVID-19 vaccine has a good effect. The effectiveness of the vaccine at one dose was 77.0 %, and at two doses, it was 86.7 %, as demonstrated by our results (Bruxvoort et al., 2021). A further study by Tan et al. showed that hospitalization with Delta and Omicron variations was significantly prevented by the three-dose vaccination with mRNA-1273 (Tan et al., 2023).

## 7 Conclusion

In conclusion, this systematic review provides initial evidence supporting the effectiveness and safety of COVID-19 vaccine boosters in immunosuppressed individuals, helping to enhance their immune responses against SARS-CoV-2. Nonetheless, further research is necessary to determine the optimal timing intervals between primary vaccination and boosters, taking into account global equity considerations and variant-specific factors. It is crucial to integrate these findings into real-time public health strategies as new data emerges. Policymakers should rely on robust scientific evidence when formulating guidelines on booster dose administration, aiming to maximize public health benefits during this ongoing pandemic. Given the low number of subjects, it is imperative to support additional studies focusing on immunosuppressed individuals who are particularly vulnerable to severe COVID-19 or other infectious diseases. By doing so, we can better protect this vulnerable population and inform future pandemic response efforts.

## Authors contribution

(1) The conception and design of the study: *Esmaeil Mehraeen*, *SeyedAhmad SeyedAlinaghi*
(2) Acquisition of data: *Arian Afzalian, Mohsen Dashti, Haleh Siami*
(3) Analysis and interpretation of data: *Esmaeil Mehraeen, SeyedAhmad SeyedAlinaghi, Omid Dadras*
(4) Drafting the article: *Afsaneh Ghasemzadeh, Sanaz Varshochi, Sahar Nooralioghli Parikhani, Masoomeh Fathi Amrollah, Anahid Nourian*
(5) Revising it critically for important intellectual content: *SeyedAhmad SeyedAlinaghi, Omid Dadras*
(6) Final approval of the version to be submitted: *SeyedAhmad SeyedAlinaghi, Esmaeil Mehraeen, Omid Dadras*

## Funding

This research did not receive any specific grant from funding agencies in the public, commercial, or not-for-profit sectors.

## CRediT authorship contribution statement

**SeyedAhmad SeyedAlinaghi:** Conceptualization, Design, Writing – review & editing. **Mohsen Dashti:** Writing – original draft. **Arian Afzalian:** Writing – original draft. **Haleh Siami:** Writing – original draft. **Afsaneh Ghasemzadeh:** Writing – original draft. **Sanaz Varshochi:** Writing – original draft. **Sahar Nooralioghli Parikhani:** Writing – original draft. **Masoomeh Fathi Amrollah:** Writing – original draft. **Anahid Nourian:** Writing – original draft. **Esmaeil Mehraeen:** Conceptualization, Writing – original draft, Writing – review & editing. **Omid Dadras:** Writing – original draft, Writing – review & editing.

## Declaration of competing interest

The authors declare that they have no known competing financial interests or personal relationships that could have appeared to influence the work reported in this paper.

## Data Availability

No data was used for the research described in the article.
