# Supplementary material for: The immunologic outcomes and adverse events of COVID-19 vaccine booster dose in immunosuppressed people: A systematic review
Source: Prev Med Rep. 2024 May 31;44:102778. doi: 10.1016/j.pmedr.2024.102778 (PMC11228787; doi:10.1016/j.pmedr.2024.102778)
Supplement: Supplementary data [file mmc1.docx]

**Table 1 –**Description of the findings reported in eligible studies

| **ID** | **Country/Year** | **Number of participants** | **Study design** | **Characteristics of participants** | **Vaccine type** | **Number of vaccine doses** | **Vaccine response assessment Method** | **Vaccine response assessment Onset** | **The interval between vaccine doses** | **SARS-CoV-2 Variants** | **Adverse events** | **Vaccine** **efficacy  (Humoral and/or Cellular response)** | **Main findings** |
| --- | --- | --- | --- | --- | --- | --- | --- | --- | --- | --- | --- | --- | --- |
| 1 | Germany  2022 | 309 hemodialysis (HD)  Patients and 36 kidney transplant recipients (KTR) | Retrospective observational | Median age: 68 years  Male: 61.5% | mRNA-1273 (Moderna)  BNT162b2 (BioNTech/Pfizer) | 309 HD patients received the 3^rd^, and 182 HD patients received the 4^th^ dose. | SARS-CoV-2 receptor-binding domain (RBD) IgG titers  Whole blood interferon-γ (IFN-γ) | 40 days after third dose  34 days after the fourth dose. | 188 days after the 2^nd^ for the 3^rd^ dose.  309 days after the 2^nd^ dose for the 4^th^ dose. | N/A | N/A | The median IgG titers in HD patients after 4^th^ dose was 6923.5 BAU/mL and significantly higher in comparison to the 3^rd^ vaccination (2882.70 BAU/mL)  HD patients receiving four doses of the Moderna vaccine had higher  IgG levels than patients vaccinated with four doses of Pfizer. | The highest antibody titers among HD individuals were induced by cross vaccination using  both Moderna and Pfizer.  Only 10 out of 29 (third dose) KTX patients had a measurable  humoral immune response after three doses. |
| 2 | Denmark  2022 | 395  solid organ transplant (SOT)  (Kidney, Liver, Heart, Lung, and combined) | Prospective cohort | median age: 58.2 years   Male: 58.5% | BNT162b2 (BioNTech/Pfizer) | 3 doses. | Anti Spike protein IgG antibodies | 72 days after the 2^nd^ dose and 40 days after the 3^rd^ dose. | A median of 231 days after the 2^nd^ dose. | N/A | N/A | The SARS-CoV-2 spike S1 IgG antibodies were found in 49.4% of participants after the 2^nd^ dose and 77.9% of patients after the 3^rd^ dose.  Among the 200 seronegative patients after the 2nd dose, 47.5% became seropositive after the 3^rd^ dose, while 36.5% remained negative.  47.5% of participants demonstrated at least a 10-fold increase in antibody concentrations after the 3^rd^ dose. | The increase in antibody titers was significantly higher among patients with detectable antibodies after the 2^nd^ dose when compared to those without.  A longer time since transplantation and liver recipients were positively associated with an antibody response while increased age, prednisolone administration, and proliferation inhibitors were associated with a reduced antibody response. |
| 3 | Spain  2022 | 36 patients with solid tumors | prospective observational | Mean age: 59.36  Male: 33.3% | mRNA-1273 (Moderna) | 3 doses | SARS-CoV-2 receptor-binding domain (RBD) IgG titers  IFN-γ | Two months after the 2^nd^, and two months after the 3^rd^ dose. | N/A | N/A | N/A | Median IgG titers after the 2^nd^, and the 3^rd^ dose was 4,914 UA/mL and 25,541 UA/mL  Serological conversion occurred in all patients after the 2^nd^ dose, with significantly higher titers than baseline.  higher antibody titers were also seen between the 1^st^ and 3^rd^ and the 2^nd^ and 3^rd^ vaccine doses.  Cellular response was observed in 36.1%, and 91.7% of patients after the 1^st^, and 2^nd^ dose, respectively. | 66.7% of patients who had partial specific IFN-γ response after the 2^nd^ dose that was further enhanced after the 3^rd^ dose, while 30.5% demonstrated a significant fall-off of specific IFN-γ production after the 3^rd^ dose. |
| 4 | France 2022 | 67 kidney transplant recipients | Prospective cohort | Median age: 56.6 years   Men: 61.2% | mRNA-1273 (Moderna) | 4 doses | Anti-SARS-CoV-2 spike receptor-binding domain (RBD) IgG titers | >1 month | Median of 68 days after the 3^rd^ dose | Delta variant | N/A | After the 4^th^ dose, the median anti-RBD titer increased significantly from 13 (BAUs)/ml to 112.5 BAUs/ml.  The proportion of patients who had neutralizing antibodies against the Delta strain rose from 16% to 66% before and after the 4^th^ dose. | 81% of patients with a weak immune response after the 3^rd^ dose showed a strong anti-RBD IgG response after the 4^th^ dose. |
| 5 | Norway  2023 | 536 patients received the 4th dose (Vaccine group)  167 participants had COVID-19 after the 3^rd^ vaccine dose (Hybrid group)  149 healthy control who received the 3^rd^ dose.  (Control group) | Prospective observational | All patients in vaccine or hybrid group had some form of immune-mediated inflammatory diseases:  rheumatoid arthritis, spondyloarthritis, psoriatic arthritis, Crohn's disease, or ulcerative colitis.  Median age and Male (%): Vaccine group: 59 years 43% Hybrid group: 44 years 46% Control group: 45 years 12% | BNT162b2 (BioNTech/Pfizer)  mRNA-1273 (Moderna)  ChAdOx1 nCoV-19 vaccine (AstraZeneca) | 3 to 4 doses (4^th^ doses were all mRNA vaccines) | The receptor-binding domain of SARS-CoV-2 spike protein (anti-RBD antibodies) | 2–4 weeks after the 3^rd^ or 4^th^ dose in the vaccine group, hybrid group, and the healthy control group. | In vaccine group: The median of 106 days after the 3^rd^ dose. | N/A | No serious adverse events were reported.  Disease flare was reported in 7% of 491 patients following the 4^th^ vaccine dose. | In the vaccine group, anti-RBD antibody titers were significantly higher after the 4^th^ dose than the 3^rd^ dose but were significantly lower than in the healthy control group vaccinated with three doses.  In the vaccine group, higher anti-RBD antibody titers after the 4^th^ dose compared to the 3^rd^ dose were found across all diseases.  In the hybrid group, anti-RBD antibody titers were significantly higher than in the vaccine group.  Antibody titers in the hybrid group were also significantly higher than in the control group. | Tumor necrosis factor (TNF) inhibitor, and rituximab treatment, were associated with lower humoral immune responses after the 4^th^ dose than the reference methotrexate. |
| 6 | Austria  2022 | 147 kidney transplant recipients who were seronegative after 2 vaccine doses. | Retrospective cohort | Median age: 60.6 years  Male: 66.9% | BNT162b2 (BioNTech/Pfizer)  mRNA-1273 (Moderna) | 3 to 4 doses. | The receptor-binding domain of SARS-CoV-2 spike protein (anti-RBD antibodies) | 37 days after the 3^rd^ and 26 days after the 4^th^ dose. | N/A | N/A | One patient developed acute kidney rejection after 2 weeks of the 3^rd^ dose.  One other patient (with a history of non-adherence to immunosuppression) developed rejection after 4 months of the 2^nd^ dose. | The seroconversion seronegative patients were 51.54% of (n = 324) patients after a 2^nd^ dose, 63.95% of (n = 147) seronegative patients after a 3^rd^ dose, and 29.27% of (n = 41) seronegative patients after a 4^th^ dose. | The cumulative prevalence of seropositivity was 51.5% after two doses, 80.5% after three doses, and 84.2% after four doses. |
| 7 | Italy  2022 | 25 Solid organ transplant recipients (SOTRs) | Prospective Cohort | Median age: 55 years  Male: 64% | BNT162b2 (BioNTech/Pfizer)  mRNA-1273 (Moderna) | 15 received the fourth dose, and 10 contracted COVID-19 after the 3^rd^ dose. | Anti Spike protein IgG antibodies  IFN-γ-secreting T cells, | two months after the 4^th^ dose. | 4^th^ dose is 168 days after the 3^rd^ dose.  Contracted SARS-CoV-2 infection 134 days after the 3rd dose. | N/A | N/A | A significant increase by three-fold in Anti Spike protein IgG titers was observed after the 4^th^ dose compared to post-3rd dose levels.  Among patients recovered from COVID-19 a 10.5-fold significant increase in Anti Spike protein IgG titers was observed. | Among the 15 SOTRs who received the 4^th^ dose no significant difference in T-cell responses was observed before and after the 4^th^ dose.  Interestingly, in patients recovered from COVID-19 a 2.5-fold significant increase in Spike-specific T-cell response was observed before and after the COVID-19 infection. |
| 8 | Italy  2022 | 45  kidney transplant recipients | Prospective cohort | Mean age:  52.6 years  Male: 51.1% | BNT162b2 (BioNTech/Pfizer) | 3 doses | Anti-Spike IgG Anti-Bodies  SARS-CoV-2 neutralizing antibody titer (NAbs)  Spike-specific T-cell response  anti-HLA and anti-DSA antibodies (for transplant rejection determination) | 21 days and 3 months after the 3^rd^ dose. | >6 months | N/A | N/A | Six months after the 2^nd^ dose, total IgG, SARS-CoV-2 NAbs and Spike-specific T-cell response prevalence rates were 45%, 38%, and 59.5%, respectively.  One month after the 3^rd^ dose total IgG, SARS-CoV-2 NT Abs, and Spike-specific T cell response prevalence rates increased to 53%, 60%, and 75.6% respectively. After two doses, total anti-Spike IgG was 6.9 BAU/mL after six months. After the 3^rd^ dose, total anti-Spike IgG peaked at 52.5 BAU/mL three weeks after the 3^rd^ dose and decreased to 41.4 BAU/mL at three months after the 3^rd^ dose.  Spike-specific IFNγ-producing T cells rose from 7.5 at six months post-2nd dose to 25 after the 3rd dose. | 24.4% of patients reported SARS-CoV-2 infection after administration of the third dose, while there were no differences in SARS-CoV-2 immune response caused by BNT162b2 vaccination between infected and uninfected subjects after the 3^rd^ dose.  HLA antibodies were recorded 15.5% patients, but none of the patients developed acute renal rejection. |
| 9 | Belgium  2022 | 49 Lung transplant recipients | prospective and non-interventional clinical trial | Median age: 63  female: 48.9% | A two-dose regimen with ChAdOx1 nCoV-19 vaccine (AstraZeneca) followed by a 3^rd^ heterologous BNT162b2 (BioNTech/Pfizer) | 3 doses | Anti-spike S1 SARS-CoV-2 IgG  neutralizing capacity of antibodies (NAbs) | Samples were collected within 28, 84, 112, 180, 210, and 238 days after the first dose. | 18 weeks | wild-type, Delta and Omicron | N/A | Two doses of ChAdOx1 nCoV-19 induced poor immunogenicity with 7.2% seropositivity at day 180 and low neutralizing capacities.  The 3^rd^ dose induced significant increases in IgG titers and neutralizing antibodies.  The 3^rd^ dose caused a significant increase in mean NAbs titers at day 238 when comparing with primary vaccination and increased by 9.5-fold between day 210 and 238.  Seroneutralization capacities against Delta and Omicron variants were found in only 13 and 9 LTRs, respectively. | Two patients (4.0%) were considered responders after the 1^st^ dose.  Among 42 seronegative patients after the 1^st^ dose, only one seroconverted after the 2^nd^ dose.  two-dose regimen of AstraZeneca vaccine caused poor immunogenicity as 92.8% of patients failed to form antibody response while, a 3^rd^ dose BNT162b2 vaccine, resulted in higher rates of both seroconversion and positive NAbs against in 32.2% of triple-vaccinated patients.  Mycophenolate mofetil and high-dose corticosteroids were associated with a weak serological response. |
| 10 | Germany 2022 | 243 Cardiothoracic transplant recipients (228 heart, 14 lung, 1 heart–lung recipients) | prospective single-arm trial | Median age: 62 years  females:25% | BNT162b2 (BioNTech/Pfizer) | 3 doses | Neutralizing antibody capacity (NAC)  Anti-SARS-CoV2-immunoglobulins (IgGs) | NAC and IgGs were measured: prior to booster vaccination (T1) 28 (T2), 56 (T3) days, and in case of a positive response at T2 120 days (T4) thereafter. | 141 days following the 2^nd^ dose. | N/A | N/A | Sero-positivity was found in 129 patients (53%) at T2. The remaining 114 patients remained sero-negative.  of 129 seropositive patients after the 3^rd^ dose (T2), 73 patients had no detectable IgG-titres prior to the 3rd booster dose.  the remaining 56, sero-positive patients at T2, had weak, but detectable IgG-titres at T1, and both the humoral response and NAC was more pronounced among them when compared to 73 seronegative patients after the 3rd dose at T2. | All 15 lung recipients were non-responders.  Non-responders were more frequently on triple immunosuppression, especially on the combination of tacrolimus, mycophenolate, and prednisone. the 3rd dose vaccine causes seropositivity in a proportion of cardiothoracic transplant recipients, whereas almost half of patients still lack a humoral immune response. |
| 11 | USA 2023 | 43 kidney transplant recipients | retrospective observational | Median age: 18 years   Male: 61% | BNT162b2 (BioNTech/Pfizer)  mRNA-1273 (Moderna) | 3 doses | SARS-CoV-2 anti-spike protein (anti-S) | 56 days after dose 2 and 39 days after dose 3. | N/A | Delta variant | there were no serious adverse events reported after vaccination. | In those responding only after a 3rd dose, anti-S titer rose significantly from 9.4 AU/mL to 682 AU/mL versus an increase from 4.9 AU/mL to 7.4 AU/mL in those who did not respond to the 3^rd^ dose.  Seroconversion was noted in 56% of 2-dose recipients and increased to 85% in those who received a 3^rd^ dose. | Among patients who did not seroconvert after the 2^nd^ dose, 75% seroconverted following the 3^rd^ dose.  Anti-S titers were significantly higher in those who received a 3^rd^ dose. |
| 12 | Israel 2022 | 73 Liver Transplant adults (23 received 3 doses, and 50 received the 4^th^ dose) | Cohort | Age median: 63.8 years  Male:  51% | BNT162b2 (BioNTech/Pfizer) | 3 or 4 doses | IgG antibodies against the SARS-CoV-2 spike RBD | 132 days after the 3^rd^ vaccine and 29 days after the 4^th^ dose. | A median of 38 days after 3^rd^ vaccine follow-up serology assessment. | Wild-Type and Omicron | Adverse effects were reported by 25%; including shoulder pain, muscle pain, headache, and fatigue. | Significant differences in 3^rd^ vs 4^th^ dose: Receptor-binding domain (RBD) IgG: 345 (BAU/mL)  vs.  2118 (BAU/mL).  Omicron BA.1 and BA.2 neutralizing antibodies 3^rd^ vs 4^th^ doses: (10 vs. 87), and (15 vs. 149) respectively. Positive Ab responses were higher in 1 and 3-month follow-ups among 4^th^ dose recipients compared to 3-dose recipients (83.3% vs. 75%) and (69.6% vs. 81.6%) | Breakthrough infections were documented among 18% of 4^th^ dose recipients vs. 30.4% of 3^rd^ dose recipients.   the 4^th^ dose significantly improved humoral immune responses among Liver Transplant recipients. |
| 13 | Greece 2022 | 39 CLL patients. | prospective, non-interventional | Median age: 73 years  Male: 43.6% | BNT162b2 (BioNTech/Pfizer) | 3 doses | anti-SARS-CoV-2 receptor binding domain (RBD) spike protein IgG (anti-RBD). | median of 14 days. | A median of 5.6 months after the 2^nd^ dose. | N/A | N/A | The seroconversion rate rose from 28.2% before the 3^rd^ dose to 64.1% after the 3rd dose and it was higher in treatment-naïve patients.  eight patients who did not achieve a seroconversion after the 2^nd^ dose (38.1%), did so after the 3^rd^. | the use of venetoclax is correlated with higher immunogenicity/seroconversion rates than that of ibrutinib. |
| 14 | USA 2022 | 28 Heart Transplant patients, of whom 12 received the 3^rd^ dose. | Prospective observational | Median age at the 1^st^ dose: 16.5 years.  Male: 64% | mRNA COVID‐19 vaccine | 3 doses | SARS‐CoV‐2 IgG spike protein | 34 days | 97.5 days after the 2^nd^ dose. | N/A | After the 2^nd^ and 3^rd^ sore arm and fatigue were most prevalent. | Ab production after 3^rd^ dose was observed among four of seven (57%) recipients who were negative after their second dose.  61% of recipients had detectable Abs at a median of just over 3 months from second‐dose vaccination. | No myocarditis, acute rejection, graft dysfunction, graft loss, or deaths were observed. |
| 15 | UK 2022 | 80 patients with blood cancer | Cohort | Mean Age: 63 years Male: 59% | BNT162b2 (BioNTech/Pfizer) | 4 doses | Neutralizing antibody titers (NAbTs) | 28 days after the 3^rd^ dose and 18 days after the 4^th^ dose. | 92 days after the 3^rd^ dose | WT, Delta, and Omicron BA.1 and BA.2 |  | Following the 4^th^ dose, 90% of patients had detectable NAbT against BA.2, which was higher than the proportion with NAbT against BA.1.  After 3rd dose, 62%, 87%, and 72% of patients had detectable NAbT against Omicron BA.1, wild-type (WT), and Delta, respectively.  After 4^th^ dose, detectable NAbT rate against Omicron BA.1, WT, and Delta was 79%, 98%, and 78%, respectively. | Significant differences in the rate of patients with detectable NAbT after 3^rd^ vs. 4^th^ doses were found for Omicron BA.1 and WT, but not for Delta. Patients who received B cell-depleting therapies within the 12 months before vaccination had the greatest risk of not having detectable NAbT. |
| 16 | USA  2022 | 43 people with HIV | RCT | Age: 42.5 years  Male: 43% | ChAdOx1 nCoV-19 (AstraZeneca) | 3 doses | anti-SARS-CoV-2 spike IgG  T-cell immune responses | 182 days after the third dose | 1 year after second dose | Alpha, Beta, Gamma, and Delta variants |  | Anti-SARS-CoV-2 spike IgG titers and CD4⁺ SARS-CoV-2 levels were significantly higher after the 3^rd^ dose compared to baseline levels. | there were significant increases in B- and T-cell immunity. |
| 17 | Italy  2022 | 19 myelofibrosis patients /20 healthy controls | Cohort | myelofibrosis patients  Age: 72 years  Male: 42%  Healthy controls  Age: 52 years  Male: 55% | BNT162b2 (BioNTech/Pfizer)  mRNA-1273 (Moderna) | 3 doses | Anti-SARS-CoV-2 spike IgG  ACE2/RBD inhibition binding activity  Spike-specific B cells | 15 days after the third dose | 6 months after the second dose |  |  | The 3^rd^ vaccine dose significantly boosted the anti-spike IgG response, reaching antibody levels in both myelofibrosis patients and healthy controls.  After the 3^rd^ dose, ACE2-RBD inhibition activity was observed in 88% of ruxolitinib treated and 82% of ruxolitinib untreated myelofibrosis patients. | The third mRNA vaccine dose remarkably elevated the SARS-CoV-2 specific humoral and B cell responses. |
| 18 | Greece  2022 | 58 patients with Waldenstrom Macroglobulinemia, Chronic Lymphocytic Leukemia, and Non-Hodgkin Lymphoma  213 healthy controls | Cohort | Age: 75 years  Male: 28% | BNT162b2 (BioNTech/Pfizer)  ChAdOx1 nCoV-19 (AstraZeneca) | 3 doses | Neutralizing antibodies (NAb) | 22 days after the second dose | 3 weeks after the third dose | N/A | N/A | The median NAb inhibition  titer was 17% for WM/CLL/  NHL patients versus 32%  for controls. (P<0.001) | The booster dose of both Pfizer and AstraZeneca  vaccines resulted in the production of NAbs against SARS-CoV-2 in patients with WM/CLL/NHL but to a significantly lower level compared to healthy individuals. |
| 19 | Italy  2022 | 42 HIV-infected subjects | Cohort | Median age: 53 years  Male: 37% | BNT162b2 (BioNTech/Pfizer) | 3 doses | CD4⁺ and CD8⁺ T-cell counts | 4 weeks after the third dose. | 5.54 months after the second dose |  | Minor local and systemic side effects were observed. | CD4⁺ T-cell median count rose from 687 at to 732 cells/mm3 post-3^rd^ dose vaccination.  CD8⁺ T-cell median count increased from 701 to 751 post-3rd dose vaccination. | The 3^rd^ dose of the Pfizer vaccine in PLWH  was safe and increased the CD4⁺ and CD8⁺ T-cell counts. |
| 20 | USA  2023 | 67 patients with multiple myeloma | Cross-sectional | Median Age: 70  Male: 57% | BNT162b2 (BioNTech/Pfizer)  mRNA-1273 (Moderna) | 3 doses | Anti-spike IgG antibody | 14–28 days after their third dose | N/A | N/A | N/A | The 3^rd^ dose of vaccination increased antibody levels and achieved higher levels than peak levels after the first two doses. | anti-spike IgG antibodies markedly declined between the peak level achieved following their 2^nd^ dose and just prior to the administration of the 3^rd^ dose with only a small minority (7%) maintaining clinically effective antibodies. |
| 21 | Turkey  2023 | 101 Patients with solid tumors receiving active cancer treatment  48 controls | Cohort | Cohort patients  Median age: 66 years  Male: 57%  Control  Median age: 64 years  Male: 29% | BNT162b2 (BioNTech/Pfizer) | 26 patients received 3 doses and had available blood samples. | IgG antibody titers | 42 days after the third dose | 111 days after 2^nd^ dose | N/A | N/A | The seroconversion rate increased from 46.5% to 88.5% after the third‐dose booster.  the antibody titers significantly increased with the 3^rd^‐dose compared to after the 2^nd^ dose. | Among patients undergoing chemotherapy, the seroconversion rate increased from 50% to 94%.  Among those undergoing immunotherapy, the seroconversion rate increased from 33% to 78% after the 3^rd^ dose. |
| 22 | Israel  2023 | 144 kidney transplant recipients who were Omicron infected (71 4^th^ dose recipients and 73 patients 3 doses recipients)  73 healthy control | Retrospective observational | Age: 61.5  Male: 34% | BNT162b2 (BioNTech/Pfizer) | 4 doses | RBD IgG and Neutralizing Antibodies | Three to four weeks after fourth dose | 21 days | Omicron | Local and systemic adverse events were reported in 75.7% and 37.8% of the cohort, respectively, | The response rate based on Neutralizing antibody titers rose from 78.4% immediately before the 4^th^ vaccine dose to 94.6% 3 weeks after.  the mean titers of RBD IgG and NA increased significantly after the 4^th^ dose. | the 4th Pfizer dose was effective in reducing the rate and severity of Omicron disease. |
| 23 | Israel  2022 | 99 kidney Transplant Recipients | Prospective observational | Age: 66 years  Male: 74% | BNT162b2 (BioNTech/Pfizer) | 3 doses | RBD IgG and Neutralizing Antibodies | 3 to 4 weeks after the third dose. | median time of 175 days. | N/A | Local and systemic adverse events were reported in 54.6% and 41.2% of the cohort, respectively. | The response rate rose from 32.3% before the 3^rd^ dose to 85.9% after the 3^rd^ dose.  80.6% seroconverted and 96.9% remained positive following the 3^rd^ dose with a significant increase in RBD IgG and Neutralizing Antibodies. | The rate and the intensity of response to the 3rd dose were significantly higher than those found before the booster dose. |
| 24 | USA  2022 | 25 Solid Organ Transplant Recipients | Cohort | Age: 59 years  Male: 52% | mRNA vaccine | 4 doses | Anti-nucleocapsid (anti-N), anti-RBD, and anti-S IgG antibodies  ACE-2 Neutralizing Antibody | 29 (17–38) days | 93 days after the 3^rd^ dose | Omicron | N/A | The anti-N IgG titers did not significantly differ after the 4th dose.  Anti-RBD and anti-S seropositivity increased from 56% to 84% and from 68% to 88%, respectively. | The median plasma neutralization by ACE2 inhibition significantly increased after the 4th dose for all Alpha, Beta, Gamma, and Delta.  However, plasma neutralization of the Omicron variant spike protein for all participants was low and did not increase the post-4th dose. |
| 25 | South Korea  2022 | 40 patients with early breast cancer  20 controls | prospective longitudinal | Age: 51.5 years  All were female. | BNT162b2 (BioNTech/Pfizer)  mRNA-1273 (Moderna)  ChAdOx1 nCoV-19 (AstraZeneca) | 3 doses | SARS-CoV2 antigen (spike protein, RBD, and nucleocapsid) antibodies | 21-28 days after vaccination | N/A | Delta and omicron | N/A | the control group had a 4.0-fold increase in IgG response against the SARS-CoV-2 spike compared to the baseline after the 3^rd^ dose while, the breast cancer group had a 2.5-fold increase in antibody response against SARS-CoV-2 spike compared to the baseline after the 3rd dose.  The healthy control group had a 5.2-fold rise in IgA response against the SARS-CoV-2 spike compared to the baseline after the 3^rd^ dose, while the breast cancer group had a 3.6-fold increase in antibody response. | Patients under concurrent cytotoxic chemotherapy had weaker antibody response than the non-cytotoxic treatment group and healthy controls.  The titers of binding antibodies against the Delta and the Omicron (BA1) variants were lower than the wild-type, especially among breast cancer patients. |
| 26 | USA  2023 | 140 people living with HIV  75 controls | Cross-sectional | Cohort:  Age: 58 years  Male: 95%  Control:  Age: 54.9 years  Male: 43% | two doses of mRNA vaccines or one dose of adenovirus vector vaccine | 3 doses | anti-spike IgG, anti-nucleocapsid IgG, and viral neutralization antibody | 109-112 days after booster dose | N/A | Wild type (WT), Delta, and omicron |  | Regarding WT, Delta, and Omicron variants, there was no significant difference in anti-spike IgG titers or viral neutralization responses between people living with HIV and controls. | Anti-spike IgG titers and neutralization values at a median of 112 days after a booster dose in people living with HIV with preserved CD4 counts were similar to controls without HIV. |
| 27 | Greece  2022 | 85 immunocompromised individuals | Cohort | Mean age: 62.72 years  Male: 45.9% | BNT162b2 (BioNTech/Pfizer) | 3 doses | RBD‐specific IgG titers | Four weeks after the 3^rd^ dose. | 139 days between the second and third dose | Omicron | N/A | The overall IgG titers 4 weeks after 3^rd^ dose increased by more than 35-fold | Only seven subjects were infected with the Omicron variant 2–5 months after the third dose |
| 28 | Italy  2022 | 8 people living with HIV | Cohort | Age: 58 years | BNT162b2 (BioNTech/Pfizer) | 4 doses | Anti-spike IgG  spike-specific CD4⁺ T cell | 7 days, 1 month, and 2 months after the injection | 119  days between the third and fourth dose | Omicron | N/A | Anti-spike IgG levels were above the cut-off value for all patients at all timepoints. | The spike-specific CD4⁺ T cell response was reactivated one week after the fourth vaccine dose, and on average declined at two months post-vaccination. |
| 29 | USA  2022 | 30 People with Primary antibody deficiency | Cohort | Average age: 48.4 years  Male: 10% | BNT162b2 (BioNTech/Pfizer)  mRNA-1273 (Moderna) | 3 doses | SARS-CoV-2-specific B and CD4⁺ T cell response | 7-28 days after booster dose | N/A | Omicron | N/A | Most COVID-19-naïve individuals with Primary antibody deficiency syndromes had SARS-CoV-2-specific B cells after booster vaccination, with an elevated percentage of cells present at day 150 post-boost compared to before booster levels. | Primary antibody deficiency patients had reduced IgG1+ and CD11c+ memory B cell responses after the primary vaccination series, with the defect in IgG1 class-switching which was rescued following booster doses.  Boosting also increased the SARS-CoV-2-specific B and T cell response and the development of Omicron-specific memory B cells in COVID-19-naïve patients. |
| 30 | Taiwan  2022 | 399 Immunocompromised  patients | RCT | Age ≤50 years: 78.2%  Male: 25.3% | mRNA-1273 (Moderna) ChAdOx1 nCoV-19 (AstraZeneca) | 3 doses | anti-SARS-CoV-2 spike IgG titers | 4 weeks after booster dose | N/A | N/A | N/A | Immunocompromised patients had similar anti-SARS-CoV-2 spike IgG titers 4 weeks after booster dose compared with healthy participants aged ≤50 years.  Only participants with autoimmune diseases and receiving hydroxychloroquine, low-dose steroid, methotrexate, and/or sulfasalazine had numerically lower anti-SARS-CoV-2 spike IgG titers 4 weeks after booster vaccination compared to those without. | The anti-SARS-CoV-2 spike IgG titers before booster dose were lower in AstraZeneca recipients and the titers 4 weeks after booster dose were higher in participants receiving mRNA-1273. |
| 31 | Switzerland  2022 | 20 multiple sclerosis patients treated  with anti-CD-20 | Cohort | Median age: 45.8 years  Male: 55% | BNT162b2 (BioNTech/Pfizer)  mRNA-1273 (Moderna) | 3 doses | Specific T cells  anti–SARS-CoV-2 nucleoprotein  receptor-binding domain (RBD) antibody | 30 days | 26.7 weeks after the second dose | Omicron and delta | N/A | Cytotoxic T cells, specific for the vaccine strain, Delta, and Omicron variants, were present before the booster dose among 12, 10, and 9 patients, respectively.  After the 3^rd^ dose, the number of responders increased to 15 patients for the vaccine strain and to 14 for both variants. | Before the booster dose, 11 patients were seropositive for anti-RBD antibodies, which rose to 13 patients within 1 month after the 3^rd^ dose, and antibody levels rose significantly.  Specific CD8 T cells were enhanced after the 3^rd^ dose. |
| 32 | Italy  2022 | 65 Multiple Sclerosis subjects undergoing different disease-modifying therapies  9 healthy controls | Cohort | MS patients  Median age: 40 years  Male: 44.6%  Control:  Median age: 41  Male: 44.4% | BNT162b2 (BioNTech/Pfizer) | 3 doses | SARS-CoV-2 IgG Spike titers | 21 days after the third dose | 6 months after the second dose | N/A | N/A | 100% of healthy controls and 97% of disease-modifying therapies-treated Multiple Sclerosis patients were seropositive after the 3^rd^ dose. | Humoral response after booster dose in Interferon β-1a-, Dimethyl fumarate- and Teriflunomide-treated Multiple Sclerosis patients was comparable to healthy controls, while increased in Cladribine-treated Multiple Sclerosis patients.  Additionally, the 3^rd^ dose causes a seroconversion in 100% of Multiple Sclerosis patients under Fingolimod and in 65% of those under Ocrelizumab. |
| 33 | Austria  2022 | 15  11 patients were vaccinated during a continuous state of B cell depletion (CD20-vaccine cohort).  4 only the booster dose in B cell depletion.  (vaccine-CD20-vaccine cohort) | Prospective observational | vaccine-CD20-vaccine:  mean age: 35.0 ± 11.4  45% male  CD20-vaccine:  Mean age: 49.8 years  50% male | N/A | 3 doses | SARS-CoV-2 specific IgG antibody levels to the spike receptor–binding domain (RBD) | 24 days | N/A | N/A | N/A | Antibody levels were significantly higher among patients from the vaccine-CD20-vaccine cohort compared to the CD20-vaccine cohort. | observed no correlation between B cell kinetics and  SARS-CoV-2 antibody levels. |
| 34 | Iran  2022 | 43 kidney transplant recipients | Single-arm Prospective Clinical Trial | mean age: 41.99 (11.83)  Male: 65.12% | SpikoGen booster after 2 doses of Sinopharm | 3 doses | Receptor‐binding domain (RBD) and neutralizing antibodies | 30 days | 1-3 months | N/A | injection site pain and fatigue | On Day 14, seroconversion of neutralizing antibodies was 76% in the Spikogen group versus 3% in the placebo group.  seroconversion of binding IgG against the RBD protein in the pooled group was 64% for the SpikoGen group versus 0% for the placebo group. | N/A |
| 35 | USA  2022 | 378 patients with hematologic malignancy | retrospective observational | median age: 69.7  Male: 51.3 % | BNT162b2 (BioNTech/Pfizer)  mRNA-1273 (Moderna)  Ad26. COV2. S (Johnson & Johnson) | 3 doses | SARS‐CoV‐2 Total Antibody against receptor‐binding domain (RBD) | 15.6 days | 6.4 months | N/A | N/A | Seroconversion occurred in 181 patients (48%) after the initial two doses of vaccination.  Among initial non-responders to the primary course of vaccination, seroconversion after the 3^rd^ dose occurred in 56% of patients (The seroconversion rate after the booster was similar for patients on (53%) and off (58%) active therapy.) | For those who did not make detectable antibodies after the initial vaccination, over one half (65%) were able to produce antibodies after  booster dose. |
| 36 | Israel  2022 | 90 heart transplant patients | Prospective observational | mean age: 57.2 years  Male: 68.9% | BNT162b2 (BioNTech/Pfizer) | 4 doses | receptor-binding domain (RBD) IgG and neutralizing antibodies | 16.1 ± 4.0 days | 173.4 ± 4.2 days after the third dose. | Omicron and delta | N/A | Anti-RBD IgG antibodies were detected in 54 (61.4%) and 71 (80.7%) HT patients before and after the fourth dose, respectively.  The percentages of neutralizing activity against the wild-type virus, the delta, and omicron variants increased from 48%, 47%, and 24% to 68%, 66%, and 49%, respectively. | The fourth dose induced anti-RBD IgG antibodies and a higher neutralization efficiency against the wild-type virus and the delta and omicron variants; however, neutralization efficiency  against the omicron variant was lower than against the delta variant. |
| 37 | Israel  2022 | 96 heart transplant patients | Prospective observational | median age: 61.0 years  Male: 70.8% | BNT162b2 (BioNTech/Pfizer) | 3 doses | receptor-binding domain (RBD) IgG and neutralizing antibodies  interferon-gamma (IFN-γ) T cell response. | 32.3 days ± 2.4 days | 168 ± 18 days | N/A | 67% reported at least one adverse event after the third dose. At least one local injection site and systemic reaction after the third dose was reported by 60% and 20% of the 3^rd^ dose recipients, respectively. | Before the third dose, the positive antibody response was seen in 23% of the heart transplant patients, while at 18 days following the third dose it rose to, 67% of the heart transplant patients.  The third dose elicited SARS-CoV-2 neutralization  titers >9-fold and IgG anti-RBD antibodies >3-fold of the range achieved after the two primary  doses. T-cell immunity was shown for 80% of patients after the 3^rd^ dose. | Mycophenolate use, lower eGFR, and higher C-reactive protein were independently  associated with a reduced likelihood of generating an immune response. |
| 38 | France  2022 | 825 solid-organ transplant recipients  (63.4 % had received four vaccine doses, 31.2% three doses, 5% two doses, and 0.5% one dose) | Retrospective observational | median age: 61.2 years  Male: 66.7% | 97 % received BNT162b2 (BioNTech/Pfizer) | 3 and 4 doses. | Anti-SARS-CoV-2 Spike antibodies receptor binding domain (RBD) | 122 days after the 3^rd^ dose.  31.5 days after the 4^th^ dose. | 46.5 days between 2^nd^ and 3^rd^ and 201 days between 3^rd^ and 4^th^ doses. | Alpha B.1.1.7 strain | N/A | The proportion of participants with a strong  humoral response increased significantly with the number of vaccine doses:  10.6% after the 1^st^ dose  35.1% after the 2^nd^   48.5% after the 3^rd^   and 65.1% after the 4^th^. | Liver transplant recipients had more frequently a strong humoral response after the 2^nd^, 3^rd^, and 4^th^ doses. (OR = 5.3, 3.7, and 6.6 respectively) when compared with other organ transplant recipients.  Kidney transplant recipients receiving Belatacept have a poorer, although mostly detectable response. |
| 39 | Spain  2023 | 1551 patients with hematological disorders | prospective multicenter registry-based cohort | 1250  (89%), 1070 (91%), 900 (88%), and 820 (97%) had a quantitative assessment  in BAU/mL available at 3–6 weeks, 3 months,  6 months, and 12 months after full vaccination respectively.  Median age: 63 years  Male: 56.2% | mRNA-1273 (Moderna) (910 patients)  BNT162b2 (BioNTech/Pfizer) (361 patients)  ChAdOx1 nCoV-19 (AstraZeneca) (4 patients) | 3 and 4 doses.  1284 received 3^rd^ dose  430 received 4^th^ dose | SARS-CoV-2-reactive IgG antibodies against both the nucleocapsid (N) and surface (S) proteins (anti-N and anti-S IgG, respectively) | 3–6 weeks, 3 months,  6 months, and 12 months | a 3^rd^ dose at a median of 168 days after  2^nd^ dose.  a  4^th^ dose at a median of 159 days after the 3^rd^ dose. | Alfa, Beta, Delta, and omicron | N/A | 76 patients (28.5%) developed SARS-CoV-2 infection after full primary vaccination and before the booster dose, whereas 165 (62% of breakthrough infections and 12.8% of those who received the 3^rd^ dose) and 25 (9.4% of breakthrough infections and 5.8% of those who received the 4^th^ dose) were infected after the 3^rd^ dose (before 4^th^ dose) and after the 4^th^ dose, respectively. | The level of antibody titers at any time after 2-dose vaccination is strongly linked with  protection against both breakthrough infection and severe disease, even with the Omicron SARS-CoV-2 variant. |
| 40 | Italy  2022 | 47 Common Variable Immune Deficiency  patients  And  26 healthy controls | Retrospective observational | median age: 52.3 years  Male: 43% | BNT162b2 (BioNTech/Pfizer) | 3 doses | IgG antibodies against the SARS-CoV-2 (IgG S1)  T-cell and B-cell counts  Responses | 2 weeks | 4 months | N/A | N/A | In CVID, 20% of the patients had measurable anti-S1 IgG after  immunization with two doses and 64% after the booster dose, with levels rising after the  booster dose. | CVID non-responders  showed a more complex disease phenotype characterized by bronchiectasis and autoimmune  manifestations, including autoimmune cytopenia, and a high rate of SARS-CoV-2  infection post-vaccination.  The non-responder status was characterized by low average  peripheral blood lymphocyte counts, low B cells, low naïve CD4⁺ and  CD8⁺ T cells, and central memory T cells. |
| 41 | Italy 2022 | 16 patients with22q11.2 deletion syndrome  And  24 healthy donors | observational-longitudinal | Median age: 27 years  Male: 40% | BNT162b2 (BioNTech/Pfizer) | 3 doses | Anti-SARS-CoV-2 Spike IgG and IgA | 1 week | 6 months | N/A | N/A | anti-S1 antibodies decreased over time and were significantly boosted by the 3^rd^ dose. | Infected patients had more frequently moderate to severe intellectual disability, lymphopenia, and lower CD4⁺ counts.  Despite major congenital heart diseases, COVID-19 did not impact cardiological conditions.  Immunization induced Spike-specific IgG responses and generated specific MBCs and memory T cells. |
| 42 | Spain  2022 | 164 peritoneal dialysis patients | prospective and multicentric  observational | Mean age: 62 years  Male: 69% | BNT162b2 (BioNTech/Pfizer)  mRNA-1273 (Moderna) | 3 and 4 doses.  (At 12 months, the whole cohort had received 3 vaccine doses and 44 (27%) patients  had an additional 4^th^ dose | Anti- SARS-CoV-2 spike antibodies | 64 days | N/A | N/A | N/A | Patients who had received  a 3^rd^ dose had significantly higher anti-spike antibody titers at 6 months than patients with only  two doses.  Patients who had received the 4^th^ dose had higher  anti-spike antibody titers in comparison to those having received three doses, although  these differences were not statistically significant. | The fourth dose was not associated with higher rates of positive humoral  response or to statistically significant differences in anti-spike antibody titers  as compared to three doses at 12 months. |
| 43 | Germany  2022 | 29 Kidney  Transplant patients | cohort | Mean age: 59.8 years  Male: 58.6% | BNT162b2 (BioNTech/Pfizer) | 4 doses. | Anti- SARS-CoV-2 spike antibodies  Antigen-specific B cells  SARS-CoV-2 spike protein–reactive CD4⁺ T helper cells | 32 days | N/A | N/A | N/A | On day 32 after vaccination,  76% of patients showed anti–S1 domain IgG levels above the threshold for positivity.  Anti–S1  IgA was positive in 52.2% of patients and neutralization capacity was above the threshold  71.4% of patients. | A  significant increase in humoral responders at day 32 was found in 76% of patients irrespective of previous treatment  84% in  individuals on standard calcineurin inhibitor–regimen responded, with an increase in neutralizing antibodies and occurrences of  vaccine-specific B cells and Plasmablasts. |
| 44 | Israel  2022 | 102 maintenance hemodialysis patients  22 control group | Prospective observational | mean age: 75 years  Male: 59.1% | BNT162b2 (BioNTech/Pfizer) | 3 doses  66 patients  received booster dose | Anti- SARS-CoV-2 spike-1 IgG antibodies | 2–3 weeks | N/A | N/A | N/A | hemodialysis patients in the booster group had significantly  increased IgG S1 titers.  Prior antibody titers  were positively correlated to IgG levels following the booster  dose. | There was a significant association between malnutrition-  inflammation markers and the humoral response.  Humoral  response to the booster was positively correlated  with albumin and inversely correlated  with C-reactive protein (CRP). |
| 45 | USA  2022 | 4283  individuals who take immunosuppressants (including disease-modifying antirheumatic drugs (DMARD), and glucocorticoids) | cohort | Median age: 59 years  Male: 36.1% | BNT162b2 (BioNTech/Pfizer)  mRNA-1273 (Moderna)  Ad26. COV2. S (Johnson & Johnson) | 3 doses | N/A | N/A | N/A | N/A | N/A | Fully vaccinated immunosuppressed individuals who had a booster dose had a lower incidence of SARS-CoV-2 infection compared to fully vaccinated immunosuppressed individuals who did not | The mRNA-1273 and BNT162b2 vaccines are effective in individuals who take immunosuppressants. However, individuals who are vaccinated but on immunosuppressants are still at higher risk of SARS-CoV-2 infection and COVID-19 hospitalization than the broader vaccinated population. |
| 46 | Israel  2022 | 66 immunocompromised patients  44 Immunocompetent control | prospective observational | Lymphoproliferative diseases including chronic lymphatic leukemia (CLL), multiple myeloma (MM), low and high-grade non-Hodgkin lymphoma (NHL)  Mean age: 61.7 years  Male: 49% | BNT162b2 (BioNTech/Pfizer) | 3 doses | IgG anti- Spike SARS-CoV-2 antibodies  SARS-CoV-2 neutralization assay | 37±21 days | 192±17 days | N/A | N/A | One month after the 3^rd^ dose, 80% of immunocompromised patients and all (100%) of the immunocompetent individuals developed antibody response.  One month after the third dose, IgG titers were induced 7.83 folds and 2.40 folds compared to one month after the second, in the immuno-compromised and immunocompetent groups, respectively | 26% of immunocompromised individuals were seronegative after the 2^nd^, and among them, 24% elicited antibody response after the 3^rd^ dose.  A 3^rd^ dose elicited robust humoral response, superior to the response observed following the 2^nd^ dose, among immunocompetent and immunocompromised individuals. |
| 47 | Czechia  2022 | 15 patients with Spondyloarthritis treated with interleukin-17 (IL-17) and tumor necrosis factor-alpha (TNFa)  Inhibitors | Prospective observational | Male: 100%.  Mean age: 43·27 | BNT162b2 (BioNTech/Pfizer) | 3 doses | Anti-SARS-CoV-2 IgG, the anti-nucleocapsid (NCP), anti-spike 2, and RBD antibodies virus-neutralizing | 1 month | N/A | N/A | N/A | Specific antibody titers  increased after booster vaccination from 905·6 and 409·1 U/mL to  989·7 and 1000 U/mL respectively in TNFa and IL-17 groups | Immune response persistence, immunogenicity, and safety of  the 3^rd^ booster dose of the BTN162b vaccine was observed in patients with  spondyloarthritis who were treated with TNFa and IL-17 inhibitors. |
| 48 | Thailand  2022 | 89 liver transplant patients | Prospective, longitudinal observational | Mean age: 57.8  Male: 68.5% | ChAdOx1 nCoV-19 (AstraZeneca)  mRNA-1273 (Moderna) | 3 doses | Anti-SARS-CoV-2 receptor-binding domain antibodies (anti-RBD)  Surrogate SARS-CoV-2 Neutralising Antibodies  SARS-CoV-2 specific T-cell responses | 4 weeks | 3 months | Omicron | Overall, most patients experienced minor adverse events after primary vaccination and booster  No graft rejection or severe adverse events were observed. | Seroconversion was observed in 81.3% of liver transplant patients receiving ChAdOx1/ChAdOx1/mRNA-1273 and in 94.7% of those receiving ChAdOx1/BNT162b2/mRNA-1273.  The percentages of positive SARS-CoV-2 specific cellular responses after primary vaccination and booster were 51.2% and 80.5% in the ChAdOx1/BNT162b2 group and 23.5% and 52.9% in the ChAdOx1/ChAdOx1 groups, respectively. | The booster dose substantially induced robust immunity against wild-type in  most patients but was less effective against the Omicron strain |
| 49 | Italy  2022 | 40 multiple myeloma and  pre-malignant monoclonal gammopathies. | cohort |  | BNT162b2 (BioNTech/Pfizer) | 3 | SARS-CoV-2-specific IgG antibodies  SARS-CoV-2 pseudoviruses generation and neutralization assay | 14 ± 2 days | 6 months | Omicron | N/A | heterologous booster immunization improved SARS-CoV-2 spike humoral and cellular responses in newly diagnosed MM (MMD) patients and most, but not all, MM patients with relapsed-refractory disease (MMR) patients | MMR had reduced spike-specific antibody levels and neutralizing titers after SARS-CoV-2 vaccination. |
| 50 | Switzerland  2022 | 51 metastatic solid malignancies with active treatment | prospective cohort | Median age: 69 years  Males: 73% | BNT162b2 (BioNTech/Pfizer) | 46 patients received a 3^rd^ dose. | Antibodies against SARS-CoV-2 viral proteins nucleocapsid (anti-N) and spike (anti-S)  SARS-CoV-2-specific T-cells  surrogate virus neutralization test | 4 weeks | N/A | N/A | N/A | After the booster vaccination, a significant  increase in the anti-S antibody levels and SARS-CoV-2-specific T-cells was observed.  95.7% of the study population were responders after the 3^rd^ compared to only 4.3% of non-responders _­­_ | 33% of patients had breakthrough infections, but none required hospital care or died from COVID-19.  An mRNA vaccine booster dose was able to increase humoral and cellular immune responses  in cancer patients. |
| 51 | Italy 2022 | 112 patients with at least one type of primary brain tumor under active chemotherapy or biological treatments. | Prospective Observational | Male (59.8%)   <65 years old (74.5%) | BNT162b2 (BioNTech/Pfizer) | 112 patients received the 1st dose. 100 patients received the 2^nd^ dose.  73 patients received the 3^rd^ dose. | N/A | N/A | 5 to 7 months | N/A | No adverse events were reported after the booster dose. | In total, among all patients who received vaccines, only 4 were infected by the SARS-CoV-2 without symptoms. | The COVID-19 vaccine is safe and protective for a long period after vaccination among patients with primary brain tumors. |
| 52 | USA 2022 | 2,952 hospitalized patients: 1,385 case-patients and 1,567 non–COVID-19 controls 1,875 immunocompetent and  1,077 immunocompromised patients. | Case-Control | Median age: 62 years 49%: Female 58% non-Hispanic White 36% had an immunocompromising condition. | BNT162b2 (BioNTech/Pfizer)  mRNA-1273 (Moderna) | Unvaccinated, 2, and 3 doses | N/A | N/A | ≥28 days after dose 2 for immunocompromised patients. | N/A | N/A | Among immunocompromised patients: vaccine was more effective among 3-dose recipients (20% COVID-19 case-patients) compared with 2-dose recipients (30% COVID-19 case-patients). | The vaccine efficacy against COVID-19 hospitalization among immunocompromised patients was 69% for 2 doses and 88% for 3 doses (p<0.001). |
| 53 | Greece 2022 | 193 patients with B‐cell malignancies. and  matched healthy controls. | Case-Control | Non-Hodgkin Lymphoma (n=54) Waldenström's macroglobulinemia (n=90) Chronic lymphocytic leukemia (n=49) Median age: 73 years Men (47.2%) | BNT162b2 (BioNTech/Pfizer) | 3 doses | anti-SARS-CoV-2 neutralizing antibody (NAbs) | 1 month after the third vaccination. | 3 to 6 months following the second dose. | N/A | N/A | Among all B‐cell malignancies. patients, 23.5% had Neutralizing Antibodies>50% before 3rd dose versus 77.9% one month after the 3^rd^ dose.  Improved humoral responses were observed after a third BNT162b2 dose in solid‐transplant patients. | The respective subgroup numbers for Neutralizing Antibodies≥50% at one month after 3rd dose were 81.3% for CLL, 60.6% for WM, and only 35.3% for NHL.  Antibody titers were lower among B‐cell malignancy patients compared with controls of similar age and gender at all time points.  Neutralizing Antibodies≥50% were seen only in 59.1% at one month after 3rd dose among B‐cell malignancies patients. |
| 54 | Thailand  2022 | 146 kidney transplant recipients (KTRs) | Retrospective Cohort | Mean age: 47 years male: 56% | The third dose was BNT162b2 (BioNTech/Pfizer)  or mRNA-1273 (Moderna) the 1st and 2^nd^ doses were CoronaVac inactivated vaccine and ChAdOx1 nCoV-19 (AstraZeneca) | Unvaccinated, 1, 2, and 3 doses | N/A | N/A | 116 days after the second dose. | N/A | N/A | The mortality rate was 26%, 3%, and 3% in 0 to 1 dose, 2-doses, and 3-doses recipients.  the booster dose of the vaccine significantly reduced mortality. | Pneumonia developed in 50%, 23%, and 10% in 0 to 1 dose, 2-doses, and 3-doses recipients, respectively.  Hospital admission requirement was 81%, 48%, and 12% 0 to 1 dose, 2-dose, and 3-dose recipients, respectively. |
| 55 | Spain 2022 | 18 patients who received at least a dose of rituximab (Anti-CD20) in a median of last 9.6 months and 15 healthy donors. | Prospective Observational | Median age: 61 years   female:66.66%. | mRNA-1273 (Moderna) | 3 doses | IgG antibodies against the S protein of SARS-CoV-2  Plasma neutralization activity | 1 month | Median of 124.5 day | N/A | N/A | Specific IgGs against SARS-CoV-2 were increased 2.47-fold in healthy donors and 1.53-fold in rituximab-treated patients 1 month after receiving the booster dose.  100% of healthy donors and 88.9% of rituximab-treated patients demonstrated IgGs against SARS-CoV-2 in plasma after the booster dose. | neutralizing capacity 3.78-fold (p = 0.0200) higher in healthy donors and 3.03-fold (p = 0.0381) in rituximab-treated patients. The infection rate was higher in rituximab-treated individuals (33.33%), although most of the infected patients (83.4%) developed a mild form of COVID-19.  rituximab-treated individuals benefited in the humoral, but not in the cellular, immune response after receiving a booster dose |
| 56 | France 2022 | 122  hemodialyzed patients. | Retrospective, observational | mean age: 71.1 years  men 72.5% | Pfizer BioNTech (BNT162b2) | 3 doses. | Anti–spike (S1) immunoglobulin G (IgG). | N/A | ~2 months after the 2^nd^ dose. | N/A | N/A | The humoral immune response rate was 82.9% after two doses and 95.8% after three doses.  The mean titers rose from 19.5 U/ml after two injections to 170.06 U/ml after three injections. | The titers rose significantly from 7.09 U/ml with two injections to 93.26 U/ml with three injections. the mean body mass index and serum albumin levels were significantly higher in responders than in non-responders among 2-dose recipients. |
| 57 | Poland 2022 | 49 inflammatory arthritis (IA) and 47 healthy controls (HC) | Prospective cohort | IA: Age: 53 ± 13.9 female 68%  HC age: 48.6 ± 14.1 female: 71.7% | Pfizer BioNTech (BNT162b2) | 3 doses | SARS-CoV-2 S1 antigen IgG antibodies   IFN-γ production | 4 weeks | >180 days | N/A | The frequency of adverse events did not differ between the IA and the HC patients. | After the booster dose, all participants showed an increased humoral response, but the IgG titers rose more significantly in HC compared to all IA patients.  The cellular response was significantly lower both before and after the booster dose among IA patients compared to HC. | Rheumatic disease patients show lower humoral and cellular responses post-COVID-19 booster vaccines in comparison to HC. |
| 58 | USA 2022 | 496 immunosuppressed patients | Retrospective cohort study | Female: 54% median age: 50 years | mRNA-1273 (Moderna), Pfizer BioNTech (BNT162b2), or Ad26. COV2. S (Johnson & Johnson) | 3 doses | anti-SARS-CoV-2 S1 IgG   SARS-CoV-2 interferon-gamma release assay (IGRA) | 8 and 34 days post-booster injection | N/A | N/A | N/A | 62% of patients had positive anti-S1 IgG and 71% had positive IGRA after the first 2 doses, and following booster, 69% had positive anti-S1 IgG and 73% had positive IGRA.  35% of poor humoral responders to primary vaccination, developed a significantly higher response after the booster dose. Only 5% of poor cellular responders to primary vaccination, developed a significantly higher cellular response after booster dose. | Anti-CD20 monoclonal antibodies, sphingosine 1-phosphate (S1P) receptor modulators, mycophenolate, and B cell lymphoma are associated with low humoral response after the booster dose.   S1P receptor modulators and mycophenolate are associated with low cellular response after booster dose. |
